# Using the Genomic Shortcut to Predict Bacterial Behavior

**DOI:** 10.1371/journal.pbio.0030278

**Published:** 2005-07-05

While many infectious bacteria remain outside human cells as they do their damage, others, including the various species of Rickettsia, take up residence inside. This makes them especially hard to study in vivo. In this issue, Hiroyuki Ogata and colleagues show that important clues to bacterial phenotype and pathogenicity can be learned about such an intracellular pathogen by sequencing and analyzing its genome.

The bacterium they studied was R. felis, which is carried by fleas and infects cats, dogs, and even humans. Its close relatives include species that cause the deadly human diseases of typhus and Rocky Mountain spotted fever. The researchers found that the R. felis genome includes not only the expected large circular chromosome, but also two small circular plasmids, bits of DNA carrying relatively few genes that are often transferred from bacterium to bacterium. This is the first species of Rickettsia in which plasmids have been found. While Ogata et al. have not yet proved that R. felis plasmids are exchanged between bacterial cells, the larger of the two plasmids contains several genes known to facilitate this type of exchange, called conjugation.

The bacterial chromosome itself appears to code for about 1,500 proteins, the largest so far of the sequenced Rickettsia genomes. More than 500 of these appear to be unique to R. felis. Among these are a large number of transposases, enzymes that cut and paste chromosomal DNA, whose existence correlates with the large amount of repeated DNA in the genome (about 5% of the total), and the finding that the R. felis chromosome has been rearranged many times. Indeed, the chromosome bears traces of multiple types of mobile gene elements, along with gene transfers back and forth between the chromosome and the plasmids, and acquisition of genes from other, non-rickettsial, bacteria.

The authors also scanned the genome for clues to the behavior of the bacterial cell. They found genes that in other bacteria code for pili, hair-like protrusions from the cell membrane. This clue prompted them to look for pili with electron microscopy, and they found them—two types, in fact, which appear to play roles in conjugation and cell adhesion. They also found a gene that induces polymerization of actin filaments in the host cell, suggesting that R. felis uses the host cytoskeleton to get around inside the cell, as do other Rickettsia species. [Fig pbio-0030278-g001]

**Figure pbio-0030278-g001:**
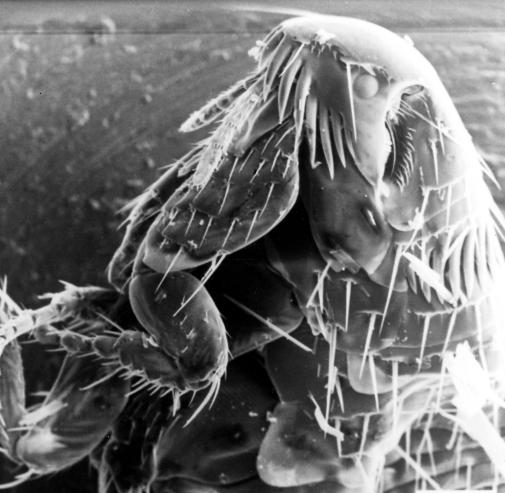
The common flea can carry Rickettsia felis bacteria in its cells

The genomic shortcut to predicting bacterial behavior may have applications in other intracellular species both in Rickettsia and beyond. Perhaps even more importantly, the discovery of plasmids in R. felis may provide a key tool for study of other Rickettsia species. Plasmids can be modified to carry other genes, and as such may offer a route for examining the biology of the more pathogenic species, including those that cause typhus, a widespread and often deadly disease.

